# Decoding the genome of multidrug resistant (MDR) *Providencia hangzhouensis* from a cerebrospinal fluid infection isolated from the hospital of Kolkata, West Bengal, India

**DOI:** 10.1128/mra.01372-25

**Published:** 2026-04-30

**Authors:** Rajsekhar Adhikary, Kusumita Acharya, Sayan Poddar, Tanmoy Sen, Mallika Ghosh, Arijit Bhattacharya, Saugata Hazra

**Affiliations:** 1 Department of Bioscience and Bioengineering, Indian Institute of Technology-Roorkee 729296 , Roorkee, Uttarakhand, India; 2 AMR-Research Laboratory, Department of Biological Sciences, Adamas University 502852, West Bengal, India; 3 Dr. Lal Path Labs-Kolkata Reference Lab, Newtown, Kolkata, India; 4 Centre for Nanotechnology, Indian Institute of Technology-Roorkee 30112, Roorkee, Uttarakhand, India; Fluxus Inc., Sunnyvale, California, USA

**Keywords:** antimicrobial resistance, broad-spectrum antibiotic resistance gene cassette, cerebrospinal fluid, multidrug Resistant (MDR), *Providencia hangzhouensis *HL_Adamas-11, whole genome sequencing

## Abstract

*Providencia hangzhouensis* HL_Adamas-11 was isolated from the cerebrospinal fluid of an infected patient. Whole-genome sequencing (WGS) revealed a 5,034,782 bp genome with 493 contigs, 49.5% GC content, and 4935 protein-coding genes. Computational prediction indicated that the beta-lactam, phenicol, macrolide, and aminoglycoside resistance gene cassette may contribute to its resistance mechanisms.

## ANNOUNCEMENT

*Providencia hangzhouensis* is an emerging Gram-negative gammaproteobacterium, often misidentified as *P. rettgeri*, causing UTIs and bloodstream infections, notable for ESBLs and carbapenem resistance (bla_IMP-27_), underscoring urgent molecular investigation for its resistance and pathogenicity (1). This study presents the genomic assembly of a multidrug-resistant *P. hangzhouensis* isolate, HL_Adamas-11, obtained from the cerebrospinal fluid (CSF) of an infected patient during the year 2023 at Dr. Lal Path Lab, Kolkata, India. Briefly, the CSF specimen was centrifuged at 3,500 rpm for 20 min. The supernatant was removed, and the sediment was vortexed to resuspend the pellet. A few drops of sediment were inoculated onto MacConkey agar and into BHI broth. MacConkey agar was incubated at 37°C for 48 h without CO2, while BHI broth was incubated for 18–24 h. Cultures were then examined for growth and turbidity to isolate the organism. The organism was subsequently subcultured in solid media to isolate. Using the standard phenol-chloroform method (2), gDNA was extracted with *A*_260_/*A*_280_=1.8 indicating quality DNA for further WGS sequencing.

To confirm the genus, the 16S rRNA gene was amplified using universal primers 8F (5′-AGAGTTTGATCCTGGCTCAG-3′) and 1492R (5′-GGTTACCTTGTTACGACTT-3′), and then Sanger sequencing was done (3). HL_Adamas-11, which was resistant to broad-spectrum antibiotics, viz. penicillins, cephalosporins, carbapenems, aminoglycosides, macrolides according to CLSI guidelines (4), had 99.93% sequence homology with *Providencia* sp. PR002 (NXKD01000055). The Illumina NovaSeq 6000 platform (Biokart Genomic Lab, Bengaluru, India) was used for whole-genome sequencing. It made 3.036 million paired-end reads (2 × 151 bp) from libraries made with the NEBNext Ultra II DNA kit (5). Using TrimGalore v0.6.10 for quality filtering ensured the average Phred score was 38 (6). SPAdes v. 2025 was used to perform *de novo* assembly, and the NCBI Prokaryotic Genome Annotation Pipeline v6.10 (7) was used to add annotations.

The draft genome had a chromosome and four plasmids. It was spread out over 493 contigs and had a total length of 5,034,782 bp with 49.5% GC content. The assembly statistics showed an *N*_50_ of 16,147 bp and a coverage of 91.664×. The CheckM analysis v1.2.4 showed that the DNA Completeness was 93.78% with Contamination percentage 5.14%. It was also predicted a total of 4,935 protein-coding sequences, with 59 tRNAs, 4 rRNAs, and 3 ncRNAs. The four plasmids had between 1 and 335 coding sequences each, and the number of contigs ranged from 1 to 25 according to PlasmidFinder v2.1 (https://cge.food.dtu.dk/services/PlasmidFinder/). The MLST ID was ST-356 according to PubMLST database (https://pubmlst.org/) with 98.75% Average Nucleotide Identity (ANI) with the reference genome. CARD v6.0.5, ResFinder v4.7.2 (8, 9) identified bla*_TEM-1_*, bla*_NDM-1_*, bla*_VEB-9_*, bla*_OXA-21_*, and bla*_OXA-181_* for β-lactam resistance. Aminoglycoside (amikacin, gentamicin, netilmicin, and tobramycin) resistance was associated with *aph(3′)-V*, *aadA21*, and *armA,* whereas *MphE*, *msrE*, *mrx*, *catA1*, and *cmlA5* were all linked to efflux-mediated resistance to macrolides and phenicols. Default parameters were used for all software except unless otherwise noted. This genome reveals extensive multidrug resistance in *P. hangzhouensis* HL_Adamas-11, warranting integrated genomic and functional studies to understand resistance evolution.

## Data Availability

16S rRNA sequence of *Providencia hangzhouensis* HL_Adamas-11 is available under GenBank accession number PX258037. The BioProject and BioSample accession numbers of the complete genome are PRJNA1314473 and SAMN51056432, respectively. The NCBI SRA accession number is SRR35282168, and the GenBank accession number is JBSGQL000000000.
